# Supplementary material for: Resolving the full spectrum of human genome variation using Linked-Reads
Source: Genome Res. 2019 Apr;29(4):635–45. doi: 10.1101/gr.234443.118 (PMC6442396; doi:10.1101/gr.234443.118)
Supplement: Supplemental Material [file supp_gr.234443.118_Supplemental_longranger-2.2.zip › longranger-2.2.tar.bz2!/lr2.2-public/lib/stan/cmdstan/stan/doc/api/html/arg__optimize_8hpp_source.html]

Stan: src/stan/gm/arguments/arg\_optimize.hpp Source File


|  |  |
| --- | --- |
|  | Stan  2.5.0  probability, sampling & optimization |


- Main Page
- Related Pages
- Namespaces
- Classes
- Files

- File List
- File Members

All Classes Namespaces Files Functions Variables Typedefs Enumerations Enumerator Friends Macros Pages

- src
- stan
- gm
- arguments

arg\_optimize.hpp

Go to the documentation of this file.

1 #ifndef STAN\_\_GM\_\_ARGUMENTS\_\_OPTIMIZE\_\_HPP

2 #define STAN\_\_GM\_\_ARGUMENTS\_\_OPTIMIZE\_\_HPP

3

4 #include <stan/gm/arguments/categorical\_argument.hpp>

5

6 #include <stan/gm/arguments/arg\_optimize\_algo.hpp>

7 #include <stan/gm/arguments/arg\_iter.hpp>

8 #include <stan/gm/arguments/arg\_save\_iterations.hpp>

9

10 namespace stan {

11

12  namespace gm {

13

14  class arg\_optimize: public categorical\_argument {

15

16  public:

17

18  arg\_optimize() {

19

20  \_name = "optimize";

21  \_description = "Point estimation";

22

23  \_subarguments.push\_back(new arg\_optimize\_algo());

24  \_subarguments.push\_back(new arg\_iter());

25  \_subarguments.push\_back(new arg\_save\_iterations());

26

27  }

28

29  };

30

31  } // gm

32

33 } // stan

34

35 #endif

36

stan::gm::arg\_optimize\_algo

**Definition:** arg\_optimize\_algo.hpp:14

stan::gm::arg\_optimize

**Definition:** arg\_optimize.hpp:14

stan::gm::arg\_optimize::arg\_optimize

arg\_optimize()

**Definition:** arg\_optimize.hpp:18

categorical\_argument.hpp

stan::gm::categorical\_argument

**Definition:** categorical\_argument.hpp:11

stan::gm::categorical\_argument::\_subarguments

std::vector< argument \* > \_subarguments

**Definition:** categorical\_argument.hpp:153

arg\_iter.hpp

stan::gm::arg\_save\_iterations

**Definition:** arg\_save\_iterations.hpp:10

stan::gm::argument::\_name

std::string \_name

**Definition:** argument.hpp:76

arg\_save\_iterations.hpp

stan::gm::arg\_iter

**Definition:** arg\_iter.hpp:10

stan::gm::argument::\_description

std::string \_description

**Definition:** argument.hpp:77

arg\_optimize\_algo.hpp


---

[ Stan Home Page ]

*© 2011–2014,
Stan Development Team.*
